# Case Report of Unusual Mortality of Egyptian Fruit Bat (*Rousettus aegyptiacus*) in northern Cyprus

**DOI:** 10.1155/crve/9140169

**Published:** 2026-07-31

**Authors:** Maya Weinberg, Dominika Kňazovická, Rianne Pelgrims, İlayda Taşkaya, Pera Sinkovec, Luis Víquez-R, Kendra Phelps, Allyson Walsh, Lucy A. Weinert, Alex J. Dumbrell, Paul A. Racey, Tigga Kingston, Julie Teresa Shapiro

**Affiliations:** ^1^ Department of Veterinary Medicine, University of Cambridge, Cambridge, UK, cam.ac.uk; ^2^ School of Life Sciences, University of Essex, Colchester, UK, essex.ac.uk; ^3^ Cyprus Wildlife Research Institute (Tashkent Nature Park), Kyrenia District, Cyprus; ^4^ Department of Biology, Bucknell University, Lewisburg, Pennsylvania, USA, bucknell.edu; ^5^ Department of Veterinary and Biomedical Sciences, College of Veterinary Medicine, University of Minnesota, St. Paul, Minnesota, USA, umn.edu; ^6^ The Bat Conservation Trust, Studio15, Cloisters House, Cloisters Business Centre, London, UK; ^7^ Department of Veterinary Medicine, Cambridge, UK; ^8^ Centre for Ecology and Conservation, Faculty of Environment, Science and Economy, University of Exeter, Cornwall, UK, exeter.ac.uk; ^9^ Department of Evolution, Ecology and Behavior, University of Illinois, Urbana, Illinois, USA, illinois.edu; ^10^ Bat One Health Working Group, Bat Specialist Group (BSG), Species Survival Commission (SSC), International Union for Conservation of Nature (IUCN), Gland, Switzerland

**Keywords:** Cyprus, mortality event, *Rousettus aegyptiacus*, *Staphylococcus aureus*

## Abstract

We report an unusual mortality event affecting the isolated Egyptian fruit bat (*Rousettus aegyptiacus*) population in northern Cyprus, combining clinical admissions, microbiological findings, and roost surveys to assess magnitude and potential drivers. From January to June 2025, the Cyprus Wildlife Research Institute (CWRI) Wildlife Hospital received an unprecedented surge in admissions with 12 individuals (compared with a total of 16 admissions from 2017 to 2024), with high acute mortality: most bats arrived moribund and died within 12–24 h. Clinical records noted localized purulent lesions and abscesses in 58% of cases. Bacteriological culture and PCR assays recovered *Staphylococcus aureus* from abscesses and skin swab samples in multiple individuals; isolates exhibited susceptibility to tested antibiotics. Necropsy sampling confirmed viable *S. aureus* in several specimens despite prolonged storage. Concurrent targeted surveys of 10 known roosts in Feb–Mar 2026 documented marked declines at multiple historical sites, including reductions from hundreds to single‐digit counts at formerly large colonies and the complete absence of bats at multiple roosts. No clear evidence of recent human disturbance, extreme weather anomalies, or reduced food availability was found; shotgun cartridges from historical hunting were present, but no direct anthropogenic cause was apparent. Although *S. aureus* infections—seasonally concentrated in winter—are consistent with observed lesions and may have contributed to morbidity and mortality, causality for the population‐level declines remains unknown, and other factors (toxins, unassessed pathogens, and multifactor stressors) cannot be excluded. Given the genetic isolation and conservation significance of the Cyprus Egyptian fruit bat population, these findings are concerning. We recommend urgent, coordinated longitudinal population monitoring, expanded pathogen surveillance including whole‐genome sequencing of *S. aureus* isolates, toxicological screening, and development of a species recovery plan incorporating emergency response, habitat protection, and public outreach.

## 1. Introduction

The Egyptian fruit bat (*Rousettus aegyptiacus*, Geoffroy 1810) is a species belonging to the family Pteropodidae. It has a widespread distribution from southern Africa, from Senegal in the west up to Egypt, the Arabian Peninsula, Levant, Türkiye, and Cyprus, and west to Pakistan [1–5]. The island of Cyprus is divided into the Republic of Cyprus in the south and the self‐declared Turkish Republic of northern Cyprus in the north—recognized only by Türkiye. The island is home to the European Union′s only population of the Egyptian fruit bat, or any pteropodid species. This island population appears isolated from the closest mainland populations in Türkiye and is thus genetically distinct [6].

Egyptian fruit bats face multiple anthropogenic threats in northern Cyprus. Starting in the 1920s, the British colonial government on the whole island carried out extermination campaigns, which were halted in northern Cyprus when it came under Turkish military control in 1974 [7]. In the Republic of Cyprus, sudden, precipitous declines in the population numbers have been observed, although the cause remains unknown [8, 9]. Although population data from northern Cyprus are more sparse than from the Republic of Cyprus [10], a major decline was observed from 2010 to 2018, with multiple roosts reduced from hundreds of individuals to mere tens and the complete abandonment of several previously occupied sites [11].

Bacterial disease appears to be another overlooked but potentially growing threat to bats [12, 13], including the Egyptian fruit bat [14]. *Staphylococcus aureus* is a common opportunistic pathogenic bacterium, although individuals may also be carriers with no signs of disease [15]. It typically acts as a secondary opportunistic pathogen, with clinical disease often developing in hosts already compromised by other primary infections or by environmental or physiological conditions that favor bacterial proliferation [16]. Nevertheless, the social structure and frequent interactions within bat colonies can facilitate rapid spread between individuals [17]. Previous studies have detected *S. aureus* in several bat species worldwide [18–20], including in Egyptian fruit bats [21, 22]. In Israel, bacterial disease in free‐ranging Egyptian fruit bats presenting severe skin lesions from which *S. aureus* was consistently isolated has been documented as a major source of morbidity [14].

Here, we describe an apparent mortality event among Egyptian fruit bats in northern Cyprus in 2025, evidenced by a marked increase in admissions to a local wildlife hospital, including individuals presenting purulent lesions for which we present bacterial findings. Simultaneously, we observed a generalized population decline in 2025, in some cases quite steep, at known roosts in the region, including caves, mines, quarries, and man‐made structures. We present current counts at these roosts and compare them with estimates from previous observations.

## 2. Case Description

### 2.1. Admissions and Case Presentations

Medical admissions of Egyptian fruit bats to the Wildlife Hospital of the Cyprus Wildlife Research Institute, a local nongovernmental, nonprofit nature conservation and research organization based in Taşkent, Kyrenia (northern Cyprus), were rare before the study period (data collected since 2016). Records indicate that one individual was admitted to the hospital in each of the years 2017, 2018, three in 2019, and two in 2020, whereas no bats were recorded in 2021 or 2022, followed by five individuals in 2023 and four in 2024. None of the cases admitted to the hospital before 2025 exhibited any observable abscesses or symptoms of a clear bacterial disease.

**Figure 1:**
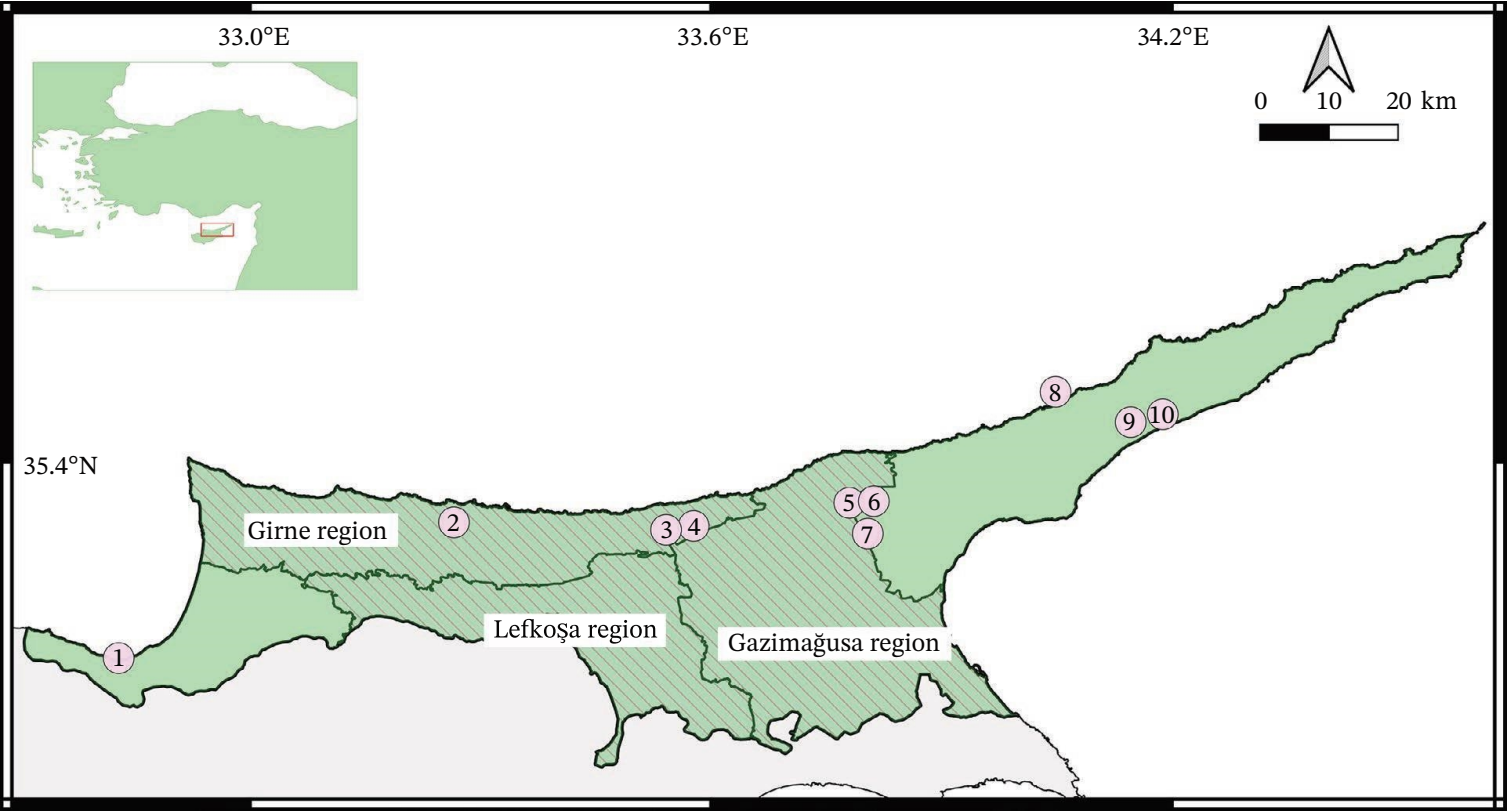
Map of northern Cyprus (light green) showing the regions of origin of the Egyptian fruit bats submitted to the Wildlife Hospital in 2025 (shaded) and locations of roosts visited during field surveys in February–March 2026 (Table 2). (1) Bağlıköy, (2) Ağırdağ, (3) Alevkaya Canyon, (4) Görneç, (5) İncirli, (6) Altınova Site 2, (7) Altınova Site 1, (8) Yedikonuk, (9) Kumyalı Site 1, and (10) Kumyalı Site 2.

In 2025, 12 Egyptian fruit bats were submitted to the Wildlife Hospital (Table 1). This is a dramatic increase, equivalent to 75% of the admissions over the previous 8‐year period in merely 6 months. Admissions occurred between January and June 2025, with a clear peak in late winter, when four bats were admitted in February and six in March, representing the highest concentration of cases over the year. The bats originated from several regions across northern Cyprus, including Lefkoşa (Nicosia; *n* = 5), Girne (Kyrenia; *n* = 6), and Gazimağusa (Famagusta; *n* = 1) (Table 1; Figure 1). As of this writing (July 2026), no Egyptian fruit bats have been admitted in 2026.

**Table 1:** Characteristics of the 12 Egyptian fruit bats (*Rousettus aegyptiacus*) brought to the Wildlife Hospital of the Cyprus Wildlife Research Institute in 2025 that were screened for bacterial infections. Two additional Egyptian fruit bats admitted in 2020 and 2024 and two Kuhl′s pipistrelles (*Pipistrellus kuhlii*) admitted in 2025 that were sampled for bacterial analysis in February 2026 are also included.

Species	Bat ID	Admission date (mm/dd/yyyy)	Region	Sex ^∗^	Age ^∗∗^	Body mass (g)	Outcome ^∗∗∗^	Abscess/lesion	Bite wounds	Bacterial screening samples (year)	*Staphylococcus aureus* isolate^a^ (year)
*Rousettus aegyptiacus*	RC1467	July 11, 2020	Kyrenia	F	A	79	DOA	No	No	Yes (2026)	No
	RC4617	November 25, 2024	Nicosia	M	A	108	DOA	No	No	Yes (2026)	Yes^+^ (2026)
	RC4642	January 1, 2025	Kyrenia	F	A	129	DOA	No	Yes	No	No
	RC4660	February 4, 2025	Nicosia	M	A	149	DOA	No	Yes	No	No
	RC4665	February 13, 2025	Nicosia	F	A	132	DOA	Yes	No	Yes (2026)	Yes
	EXTD0520	February 14, 2025	Nicosia	M	A	141	Carcass	No	No	No	No
	RC4667	February 16, 2025	Nicosia	M	A	142	D24H	Yes	Yes	Yes (2025, 2026)	Yes^+^ (2025, 2026)
	RC4681	March 8, 2025	Kyrenia	F	A	118	DBE	Yes	Yes	Yes (2025, 2026)	Yes (2025)
	EXTD0521	March 15, 2025	Nicosia	M	A	136	Carcass	Yes	Yes	Yes (2025, 2026)	Yes (2025)
	EXTD0524	March 17, 2025	Kyrenia	N/A	A	N/A	Carcass	N/A	No	Yes (2026)	No
	RC4690	March 17, 2025	Kyrenia	F	A	108	DOA	Yes	Yes	Yes (2025, 2026)	Yes (2025, 2026)
	RC4691	March 20, 2025	Famagusta	F	A	117	D24H	Yes	Yes	Yes (2026)	No
	EXTD0526	March 26, 2025	Kyrenia	F	A	112	Carcass	No	Yes	Yes (2026)	No
	RC4930	June 25, 2025	Kyrenia	F	A	110	DOA	Yes	No	Yes (2026)	Yes^+^ (2026)
*Pipistrellus kuhlii*	RC4871	May 29, 2025	NA	M	J	1.1	EUE	No	No	Yes (2026)	No
	RC5021	September 16, 2025	NA	N/A	Adult	3.0	DOA ^∗^	No	No	Yes (2026)	No

^a^ *Staphylococcus aureus* identification of isolate confirmed by whole genome sequencing (2026 isolates only).

^∗^Sex: F, female; M, male.

^∗∗^Age: A, adult; J, juvenile ^∗∗^.

^∗∗∗^Outcome categories: DOA, dead on arrival; DBE, died before examination; D24H, dead within 24 h after admission; EUE, euthanized upon examination; carcass, received as decomposing carcass.

**Figure 2:**
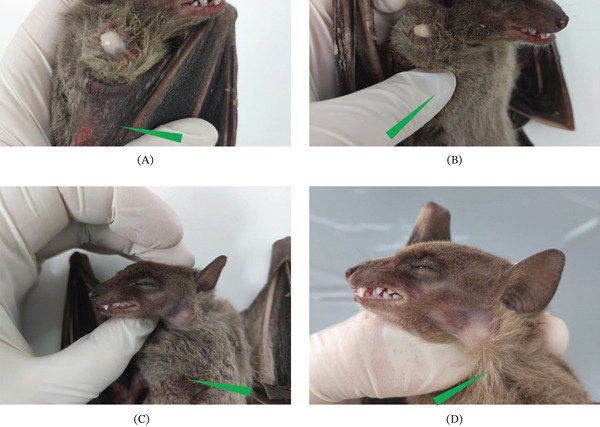
(A–B) Individual (Bat ID: RC4667) presented with an abscess on the right shoulder. (C–D) Individual (Bat ID: RC4691) presented with an abscess on the left side of the upper neck. These abscesses are the typical clinical appearance of bacterial infection with purulent (pus‐filled) content. *Source:* Photographs provided by Rianne Pelgrims, March 2025.

Of the 12 admitted Egyptian fruit bats, four were deceased at the time of initial reporting via a call to the rescue hotline, five died before arrival at the Wildlife Hospital, and the remaining three died within 24 h of admission. Seven were females and four were males, while the sex of one individual could not be determined. The average body mass of males was 142 g, whereas females averaged 118 g, which aligns with the normal body weight range and sexual dimorphism between males and females of the species in this region [26]. All 12 individuals were classified as adults in the clinical records based on their size and by examining ossification of the epiphyseal joints by shining a light behind a finger joint in the wing [27]. All bats had empty stomachs on arrival, which is typical for this species given their rapid digestion [28–30] and not indicative of pathology. Holes in the patagium were observed in three individuals, and visible wounds (resembling bite wounds) were observed on the torso and wings of eight individuals. Bite wounds were assessed as perimortem in five cases and possibly postmortem in three cases, likely inflicted by free‐ranging or feral cats [31], which are widespread in the area. In all cases, the wounds were minor and were not considered a likely or significant contributor to death in any of the bats, although they could indicate a weakened state, allowing the bats to be predated by cats. Abscesses were recorded in seven bats (58%), indicating the presence of localized purulent lesions (Table 1; Figure 2).

During the peak admission period (January–March 2025), purulent discharge was drawn from cervical abscesses of four bats for bacterial culture on discriminant plates and antimicrobial susceptibility testing via minimum inhibitory concentration (MIC) using the VITEK 2 system (bioMérieux) [32] (Table 1). The cultured bacteria were identified as *S. aureus* via visual observation of cultures, the catalase test [33], and identification with the VITEK 2 system [32]. No detectable antimicrobial resistance was found. The isolates were not retained and could therefore not be further characterized. All other bacteriological analyses were carried out in 2026 on frozen bat carcasses.

No written consent has been obtained from the subjects of this case series because all subjects are wild bats.

### 2.2. Bacteriological Analyses

The Wildlife Hospital maintains bat carcasses frozen at −20°C at their facilities. On February 18, 2026, a total of 11 Egyptian fruit bats were thawed for examination and sampling for bacteriological analyses. Nine of these bats were admitted to the hospital in 2025, one in 2020, and one in 2024 (Table 1). For the bats admitted in 2025 (*n* = 9), four of them had already been sampled for bacterial culture and antimicrobial susceptibility testing in 2025 and yielded isolates as described above. However, because these isolates had not been preserved, these individuals were sampled again in 2026.

The sampling in February 2026 involved taking fur and skin swabs and swabs from abscesses or lesions. Tissue or cavity samples from internal organs such as the lung, heart, liver, kidney, spleen, and intestinal tract were also taken for future virological and fungal analyses. In addition, the carcasses of two Kuhl′s pipistrelles (*Pipistrellus kuhlii*), an insectivorous vespertilionid species, admitted in 2025, were sampled for both bacteriological analyses and future virological and fungal analyses as an interspecies comparison (Table 1).

To culture and isolate any bacteria in the lesions, all swabs were inoculated into 10 mL of 6% NaCl enrichment broth and incubated statically at 37°C for 24 h. Our protocol targeted *S. aureus* because the appearance and location of lesions and abscesses were similar to those previously observed in infections caused by this bacterial species in Egyptian fruit bats in Israel [14]. Cultures were then mixed to homogenize them in the liquid, and aliquots (ranging from 5 to 10 *μ*L, depending on optical density) were plated onto Brilliance Staph 24 agar discriminative plates and incubated at 37°C for 24 h. Single colonies from positive plates were subcultured onto blood agar and incubated under the same conditions to ensure purity. From the blood agar plates, pure isolates were taken for molecular analysis.

In total, eight pure isolates originating from five individuals were recovered from the swabs and identified as *S. aureus*. Of these five individual bats, one was admitted in 2024 without any abscesses or lesions; the other four were admitted in 2025 and all presented with abscesses or lesions (Table 1). To confirm *S. aureus* identity, a PCR assay targeting the femB gene was performed on pure isolates. Colonies confirmed by PCR were then inoculated into 5‐mL tryptic soy broth (TSB) and incubated overnight at 37°C. From the overnight incubation of pure isolates, 1 mL of culture was centrifuged at maximum speed for 3 min to obtain a bacterial pellet. This pellet was either used for DNA extraction using the MasterPure Gram Positive DNA Purification Kit (Lucigen) or shipped as‐is to MicrobesNG (Birmingham, United Kingdom) for DNA extraction and subsequent genomic analysis.

Three pure isolates, each from a different individual bat, were sent for whole‐genome sequencing (WGS) (Table 1) with results pending.

### 2.3. Population Sampling

To evaluate potential population declines concurrent with the reported increase in mortalities, we surveyed 10 known roosting sites of Egyptian fruit bats in February and March 2026. At each roost, we counted the bats present and compared their abundance with historical observations (Table 2). We noted a decline in the number of bats at all but one of these sites. Declines were particularly dramatic at Bağlıköy dropping from an estimated 500 individuals to five between 2024 and 2026. Similarly, at Yedikonuk, the number of roosting bats has declined from 800 in 2005 to 15 in 2026. At five sites where Egyptian fruit bats or signs of their presence (e.g., feces and uneaten fruit) had been previously observed, no individuals or other signs of roost occupation could be found.

**Table 2:** Overview of historical and recent visits to Egyptian fruit bat roost sites in northern Cyprus.

Site name	Date of visit	Count	Source
Bağlıköy	November 2022	500	CWRI (unpublished data)
	March 2023	500	CWRI (unpublished data)
	August 2024	500	CWRI (unpublished data)
	March 2025	4	CWRI (unpublished data)
	February 2026	5	(This study)
Görneç	January 2018	30	[11]
	May 2018	20	[11]
	October 2018	40	[11]
	February 2026	40	(This study)
Kumyalı Site 1	May 2018	Feeding roost	[11]
	November 2022	0	CWRI (unpublished data)
	February 2026	0	(This study)
Kumyalı Site 2	May 2018	20	[11]
	October 2018	6	[11]
	February 2026	0	(This study)
Yedikonuk	October 2005	800	[23, 6]
	May 2009	2	[24]
	February 2010	20	[24]
	November 2015	40	[11]
	October 2018	20	[11]
	March 2025	1	CWRI (unpublished data)
	February 2026	15	(This study)
Alevkaya Canyon	July 2015	Detected calls	[11]
	August 2016	Detected calls	[11]
	May 2018	Detected calls	[11]
	February 2026	No calls detected	(This study)
Ağırdağ	April 2005	12	[23]
	May 2009	2	[11]
	August 2009	5	[25]
	March 2026	0	(This study)
Altınova Site 1	January 2018	1	[11]
	March 2026	0	(This study)
Altınova Site 2	January 2018	20	[11]
	March 2026	20	(This study)
İncirli	June 2002	5	Anecdotal reports
	November 2022	0	CWRI, unpublished data
	February 2026	0	(This study)

## 3. Discussion

We report a marked, recent decline of Egyptian fruit bats across northern Cyprus, documented by dramatic reductions at multiple historical roosts and an unusual surge in clinical admissions to the Cyprus Wildlife Research Institute in 2025. Although the IUCN Red List classifies the Egyptian fruit bat as a species of “least concern” with a globally stable population [2], its population on Cyprus is small, vulnerable, and genetically isolated [6, 24]. The mortality event and the significant population decline across known roosts that we observed in northern Cyprus are cause for conservation concern.

Although the 12 admissions to the CWRI Wildlife Hospital may not appear extreme, it is, in fact, highly concerning and unusual given how generally rare it is for members of the public to find bats or their carcasses. In fact, none of the bats were actively collected from natural roosts or through targeted sampling; all of these bats were found by members of the public and either brought directly to the CWRI Wildlife Hospital or transported there by the rescue team. In most years, no Egyptian fruit bats are brought in, and the highest previous count was five individuals. Generally, bat cadavers are rarely found by the public since most die in inaccessible locations or are either quickly scavenged or decompose [34, 35]. In this context, and combined with the steep declines at known roost sites, the admissions likely represent only a very small fraction of the total mortalities in this Egyptian fruit bat population.

The precise cause—or causes—of the observed mortality and population declines remain unknown. Clinical records and necropsy sampling from the Egyptian fruit bats brought to the CWRI Wildlife Hospital revealed frequent localized purulent skin lesions and abscesses located primarily in the neck and upper back. This is consistent with previously documented *S. aureus* infections in Egyptian fruit bats in Israel during the winter months, coinciding with the period of highest physiological stress [14]. Although Weinberg et al. [14] did not report mortality associated with the infections, it is plausible that severe cases could progress to death if followed longitudinally. Further, the isolation of *S. aureus* from abscesses, which are not optimal for long‐term bacterial preservation, suggests a substantial bacterial burden among the affected individuals. Although it is generally thought that *S. aureus* infections contribute to morbidity as an opportunistic pathogen but are unlikely to be the root cause of mortality (instead indicating a generally weakened state due to other factors) [14], severe *S*. *aureus* infections have been observed to cause death or debilitating injuries that impede flight in vampire bats in captivity [17]. At this time, we are unable to rule out these infections as a possible cause of death.

More generally, the winter peak in admissions, adult‐biased cases, lack of clear human disturbance at roosts, and typical environmental conditions, food availability, and body mass of admitted bats argue for a biologically driven event rather than an acute, localized human action. However, other factors (e.g., toxins, unmeasured pathogens, and nutritional stress) cannot be excluded. Reports from roosts in both northern and southern Cyprus [36], including observations shared on social media, suggest a similar peak in mortality during the same winter period across the island, indicating a potentially comparable pattern across the island and demonstrating that bats did not simply migrate to another area of Cyprus.

**Figure 3:**
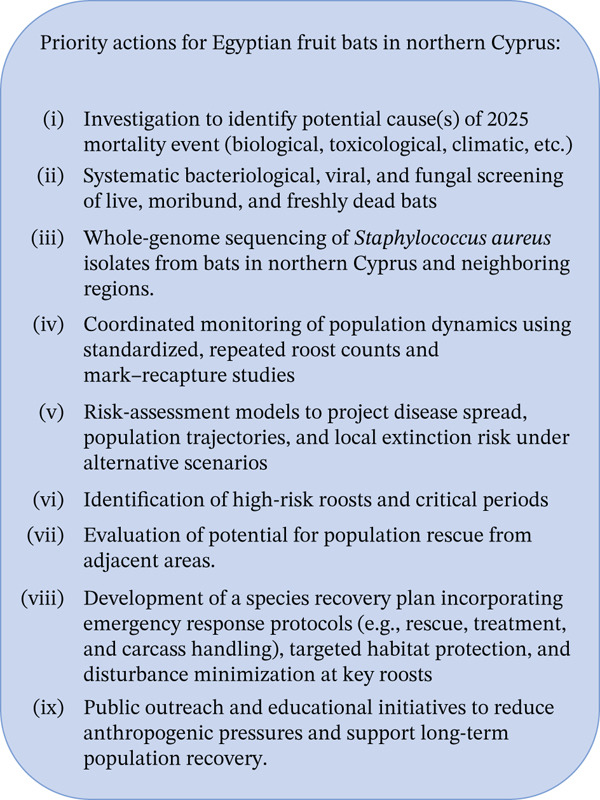
Priority action items for conservation of the Egyptian fruit bat (*Rousettus aegyptiacus*) in northern Cyprus.

Given the uncertainties regarding the mortality event and population decline of Egyptian fruit bats in northern Cyprus, urgent, coordinated investigations and monitoring are needed to elucidate the cause of this mortality event, inform effective countermeasures (Figure 3), and prevent a devastating extirpation of this species on the island [36]. Future work in the region should adopt an integrated, longitudinal framework that combines disease surveillance, ecological monitoring, and applied conservation. Pathogen surveillance should be expanded through systematic bacteriological, viral, and fungal screening of live, moribund, and freshly dead bats, with particular emphasis on WGS of *S. aureus* isolates to compare strains from healthy and symptomatic individuals and across neighboring regions. In parallel, coordinated follow‐up of population dynamics using standardized, repeated roost counts, alongside mark–recapture studies, would enable robust estimates of population size, demographic structure, reproductive success, and seasonal trends. These datasets should be leveraged to develop risk‐assessment models that project disease spread and population trajectories under alternative scenarios, including pathogen‐driven mortality and multifactor stressors. Such analyses would help identify high‐risk roosts and critical periods, estimate local extinction risk, and evaluate the potential for population rescue from adjacent areas. Ultimately, this knowledge should inform the development of a species recovery plan incorporating emergency response protocols (e.g., rescue, treatment, and carcass handling), targeted habitat protection and disturbance minimization at key roosts, and public outreach initiatives aimed at reducing anthropogenic pressures and supporting long‐term population recovery.

## Funding

This work was not funded by any specific grants or funding.

## Disclosure

An earlier version of this work was posted as a preprint on bioRxiv [37]. M.W. was supported by EU Horizon Europe under the Marie SkÅ‚odowskaâ€“Curie COFUND Grant No: 101081327 YUFE4Postdocs.

## Ethics Statement

No written consent has been obtained from the subjects of this case series because all subjects are wild bats.

## Conflicts of Interest

The authors declare no conflicts of interest.

## Data Availability

All data are included in the main text of the manuscript.

## References

[1] Bergmans W. , Taxonomy and Biogeography of African Fruit Bats (Mammalia, Megachiroptera). 4. The Genus *Rousettus* Gray, 1821, Beaufortia. (1994) 44, no. 4, 79–126.

[2] Korine C. , IUCN Red List of Threatened Species: *Rousettus aegyptiacus* , 2016, http://www.iucnredlist.org/en.

[3] Kwiecinski G. G. and Griffiths T. A. , Rousettus egyptiacus , Mammalian Species. (1999) 611, 1–9, 10.2307/3504411.

[4] Monadjem A. , Montauban C. , Webala P. W. , Laverty T. M. , Bakwo-Fils E. M. , Torrent L. , Tanshi I. , Kane A. , Rutrough A. L. , Waldien D. L. , and Taylor P. J. , African Bat Database: Curated Data of Occurrences, Distributions and Conservation Metrics for Sub-Saharan Bats, Scientific Data. (2024) 11, no. 1, 10.1038/s41597-024-04170-7, 39622813. PMC11611906 39622813

[5] Strachinis I. , Kalaentzis K. , Katsiyiannis P. , and Kazilas C. , First Record of the Egyptian Fruit Bat, *Rousettus aegyptiacus* (Pteropodidae), From Kastellorizo Island, Greece, Mammalia. (2018) 82, no. 6, 611–613, 10.1515/mammalia-2017-0063.

[6] Hulva P. , Marešová T. , Dundarova H. , Bilgin R. , Benda P. , Bartonička T. , and Horáček I. , Environmental Margin and Island Evolution in Middle Eastern Populations of the Egyptian Fruit Bat, Molecular Ecology. (2012) 21, no. 24, 6104–6116, 10.1111/mec.12078, 23094994. 23094994

[7] Hadjisterkotis E. , The Destruction and Conservation of the Egyptian Fruit Bat *Rousettus aegyptiacus* in Cyprus: A Historic Review, European Journal of Wildlife Research. (2006) 52, no. 4, 282–287, 10.1007/s10344-006-0041-7.

[8] Nicolau H. , The Egyptian Fruit Bat Rousettus aegyptiacus. Geographical Distribution, Biology and Conservation in Cyprus, 2009, Department of Biological Sciences, University of Cyprus, Msc thesis.

[9] Vaglio M. A. d. , Nicolau H. , Bosso L. , and Russo D. , A First Assessment of Feeding Habits in the Fruit Bat *Rousettus aegyptiacus* on Cyprus Island, Hystrix, the Italian Journal of Mammalogy (IT). (2011) 22, no. 2, 10.4404/hystrix-22.2-4587.

[10] Baydemir N. A. , Bat Fauna of Turkey and northern Cyprus: Species Diversity, Anthropogenic Roost Disturbance and Conservation Status, Journal of International Environmental Application and Science. (2014) 9, no. 5, 590–596.

[11] Benda P. , Satterfield L. , Gucel S. , Horáček I. , Lučan R. , Charalambidou I. , and Uhrin M. , Distribution of Bats in northern Cyprus (Chiroptera), Lynx. (2018) 49, no. 1, 91–138, 10.2478/lynx-2018-0011.

[12] Evans D. R. , Griffith M. P. , Sundermann A. J. , Shutt K. A. , Saul M. I. , Mustapha M. M. , Marsh J. W. , Cooper V. S. , Harrison L. H. , and Van Tyne D. , Systematic Detection of Horizontal Gene Transfer Across Genera Among Multidrug-Resistant Bacteria in a Single Hospital, eLife. (2020) 9, e53886, 10.7554/eLife.53886, 32285801. 32285801 PMC7156236

[13] Imnadze T. , Natradze I. , Zhgenti E. , Malania L. , Abazashvili N. , Sidamonidze K. , Khmaladze E. , Zakalashvili M. , Imnadze P. , Arner R. J. , Motin V. , and Kosoy M. , Identification of a Novel *Yersinia enterocolitica* Strain From Bats in Association With a Bat Die-Off That Occurred in Georgia (Caucasus), Microorganisms. (2020) 8, no. 7, 10.3390/microorganisms8071000, 32635480. PMC7409352 32635480

[14] Weinberg M. , Mazar O. , Rachum A. , Chen X. , Goutink S. , Lifshitz N. , Winter-Livneh R. , Czirják G. Á. , and Yovel Y. , Seasonal Challenges of Tropical Bats in Temperate Zones, Scientific Reports. (2022) 12, no. 1, 16869, 10.1038/s41598-022-21076-9, 36207354. 36207354 PMC9546901

[15] Fountain K. , Barbon A. , Gibbon M. J. , Lloyd D. H. , Loeffler A. , and Feil E. J. , *Staphylococcus aureus* Lineages Associated With a Free-Ranging Population of the Fruit Bat *Pteropus livingstonii* Retained Over 25 Years in Captivity, Scientific Reports. (2022) 12, no. 1, 10.1038/s41598-022-17835-3, 35931727. PMC9355961 35931727

[16] Haag A. F. , Ross Fitzgerald J. , and Penadés J. R. , *Staphylococcus aureus* in Animals, Microbiology Spectrum. (2019) 7, no. 3, 10.1128/microbiolspec.gpp3-0060-2019, 31124433. PMC11257167 31124433

[17] Razik I. , Carter G. G. , Abou-Elias M. , and Stockmaier S. , Opportunistic Evidence of the Impact of Bacterial Infections on Social Integration in Vampire Bats, Preprint, bio Rxiv. (2026) 10.1101/2023.04.17.537180.

[18] Akobi B. , Aboderin O. , Sasaki T. , and Shittu A. , Characterization of *Staphylococcus aureus* Isolates From Faecal Samples of the Straw-Coloured Fruit Bat (*Eidolon helvum*) in Obafemi Awolowo University (OAU), Nigeria, BMC Microbiology. (2012) 12, no. 1, 10.1186/1471-2180-12-279, 23181939. PMC3554579 23181939

[19] Attaullah S. , Ali S. , Phelps K. L. , Olival K. J. , and Ullah M. , Antimicrobial Resistance in *Staphylococcus aureus* From Bats in Pakistan, Eco Health In Press. (2026) Springer, 10.1007/s10393-026-01804-7, 42301596. 42301596

[20] Olatimehin A. , Shittu A. O. , Onwugamba F. C. , Mellmann A. , Becker K. , and Schaumburg F. , *Staphylococcus aureus* Complex in the Straw-Colored Fruit Bat (*Eidolon helvum*) in Nigeria, Frontiers in Microbiology. (2018) 9, 10.3389/fmicb.2018.00162, 29487577. PMC5816944 29487577

[21] Held J. , Gmeiner M. , Mordmüller B. , Matsiégui P.-B. , Schaer J. , Eckerle I. , Weber N. , Matuschewski K. , Bletz S. , and Schaumburg F. , Bats Are Rare Reservoirs of *Staphylococcus aureus* Complex in Gabon, Infection, Genetics and Evolution. (2017) 47, 118–120, 10.1016/j.meegid.2016.11.022, 27894991. 27894991

[22] Ngoubangoye B. , Désiré O. E. , Longo-Pendy N.-M. , Boundenga L. , Dibakou S. E. , Carrel B. D. , Sauvage F. Y. , Tsoumbou T. A. , Cyr M. , Nguema Y. O. , Maganga G. D. , and Pontier D. , *Staphylococcus* Spp. and Antibiotic Resistance in Cave Versus City Bats in Gabon, Eco Health. (2026) ahead of print, May 8 10.1007/s10393-026-01796-4, 42104192. 42104192

[23] Benda P. , Hanák V. , Horáček I. , Hulva P. , Lučan R. , and Ruedi M. , Bats (Mammalia: Chiroptera) of the Eastern Mediterranean. Part 5. Bat Fauna of Cyprus: Review of Records with Confirmation of Six Species New for the Island and Description of a New Subspecies, Acta Societatis Zoologicae Bohemicae. (2007) 71, 71–130.

[24] Benda P. , Vallo P. , Hulva P. , and Horáček I. , The Egyptian Fruit Bat *Rousettus aegyptiacus* (Chiroptera: Pteropodidae) in the Palaearctic: Geographical Variation and Taxonomic Status, Biologia. (2012) 67, no. 6, 1230–1244, 10.2478/s11756-012-0105-y.

[25] Benda P. , Abi-Said M. , Bartonička T. , Bilgin R. , Faizolahi K. , Lučan R. K. , Nicolaou H. , Reiter A. , Shohdi W. M. , Uhrin M. , and Horáček I. , Rousettus Aegyptiacus (Pteropodidae) in the Palaearctic: List of Records and Revision of the Distribution Range, Vespertilio. (2011) 15, no. 1, 3–36.

[26] Albayrak İ. , Aşan N. , and Yorulmaz T. , The Natural History of the Egyptian Fruit Bat, *Rousettus aegyptiacus*, in Turkey (Mammalia: Chiroptera), Turkish Journal of Zoology. (2008) 32, no. 1, 11–18, https://journals.tubitak.gov.tr/zoology/vol32/iss1/2/.

[27] Racey P. A. , Ageing and Assessment of Reproductive Status of Pipistrelle Bats, *Pipistrellus pipistrellus* , Journal of Zoology. (1974) 173, no. 2, 264–271, 10.1111/j.1469-7998.1974.tb03136.x, 4468893. 4468893

[28] Docters van Leeuwen W. M. , The Dispersal of Plants by Fruit-Eating Bats, Gardens Bulletin; Straits Settlement. (1935) 9, 58–63.

[29] Shilton L. , Altringham J. , Compton S. , and Whittaker R. , Old World Fruit Bats Can Be Long–Distance Seed Dispersers Through Extended Retention of Viable Seeds in the Gut, Proceedings of the Royal Society of London. Series B: Biological Sciences. (1999) 266, no. 1416, 219–223, 10.1098/rspb.1999.0625.

[30] Tedman R. A. and Hall L. S. , The Morphology of the Gastrointestinal Tract and Food Transit Time in the Fruit Bats *Pteropus alecto* and *P. poliocephalus* (Megachiroptera), Australian Journal of Zoology. (1985) 33, no. 5, 625–640, 10.1071/ZO9850625.

[31] Oedin M. , Brescia F. , Millon A. , Murphy B. P. , Palmas P. , Woinarski J. C. Z. , and Vidal E. , CatsFelis catusas a Threat to Bats Worldwide: A Review of the Evidence, Mammal Review. (2021) 51, no. 3, 323–337, 10.1111/mam.12240.

[32] Ligozzi M. , Bernini C. , Bonora M. G. , De Fatima M. , Zuliani J. , and Fontana R. , Evaluation of the VITEK 2 System for Identification and Antimicrobial Susceptibility Testing of Medically Relevant Gram-Positive Cocci, Journal of Clinical Microbiology. (2002) 40, no. 5, 1681–1686, 10.1128/JCM.40.5.1681-1686.2002, 11980942. 11980942 PMC130656

[33] Koneman E. W. A. S. , Janda W. M. , Schreckenberger P. C. , and Winn W. C. , The Gram Positive Cocci: Staphylococci and Related Organisms, Color Atlas and Textbook of Diagnostic Microbiology, 1992, JP Lippincott Company.

[34] Arnett E. B. , Kent Brown W. , Erickson W. P. , Fiedler J. K. , Hamilton B. L. , Henry T. H. , Jain A. , Johnson G. D. , Kerns J. , Koford R. R. , and Nicholson C. P. , Patterns of Bat Fatalities at Wind Energy Facilities in North America, Journal of Wildlife Management. (2008) 72, no. 1, 61–78, 10.2193/2007-221.

[35] Bastos R. , Santos M. , and Cabral J. A. , A New Stochastic Dynamic Tool to Improve the Accuracy of Mortality Estimates for Bats Killed at Wind Farms, Ecological Indicators. (2013) 34, 428–440, 10.1016/j.ecolind.2013.06.003.

[36] Dechmann D. K. N. , Akre K. L. , de Wit L. A. , Kafkaletou Diez A. G. , Mandl I. , Makridou Z. , Michael K. , O′Mara M. T. , Papastylianou K. , Wikelski M. , and Frick W. F. , Rapid Population Decline of Egyptian Fruit Bats on Cyprus, 2026, Preprint, bio Rxiv 10.64898/2026.06.05.728241.

[37] Weinberg M. , Knazovicka D. , Pelgrims R. , Taskaya I. , Sinkovec P. , Viquez-R L. , Phelps K. , Walsh A. , Racey P. A. , Kingston T. , and Shapiro J. T. , Case Report of Unusual Mortality of Egyptian Fruit Bat (*Rousettus aegyptiacus*) in northern Cyprus, 2026, Preprint, bio Rxiv 10.64898/2026.06.08.730983. PMC13426321 42540694

